# IGF-1 and IGFBP-3 in Inflammatory Cachexia

**DOI:** 10.3390/ijms22179469

**Published:** 2021-08-31

**Authors:** Ana Isabel Martín, Teresa Priego, Álvaro Moreno-Ruperez, Daniel González-Hedström, Miriam Granado, Asunción López-Calderón

**Affiliations:** 1 Department of Physiology, Faculty of Medicine, Complutense University of Madrid, 28040 Madrid, Spain; anabelmartin@med.ucm.es (A.I.M.); alvmor02@ucm.es (Á.M.-R.); 2 Department of Physiology, Faculty of Nursing, Physiotherapy and Podiatry, Complutense University of Madrid, 28040 Madrid, Spain; tpriegoc@ucm.es; 3 Department of Physiology, Faculty of Medicine, Autonomous University of Madrid, 28049 Madrid, Spain; dgonzalez@pharmactive.eu (D.G.-H.); miriam.granado@uam.es (M.G.); 4 Pharmactive Biotech Products S.L. Parque Científico de Madrid, Avenida del Doctor Severo Ochoa, 37 Local 4J, 28108 Alcobendas, Spain; 5 CIBER Fisiopatología de la Obesidad y Nutrición, Instituto de Salud Carlos III, 28029 Madrid, Spain

**Keywords:** GH, IGF-1, IGFBP-3, sepsis, inflammation, muscle wasting, glucocorticoids, cytokines, nitric oxide, cachexia

## Abstract

Inflammation induces a wide response of the neuroendocrine system, which leads to modifications in all the endocrine axes. The hypothalamic–growth hormone (GH)–insulin-like growth factor-1 (IGF-1) axis is deeply affected by inflammation, its response being characterized by GH resistance and a decrease in circulating levels of IGF-1. The endocrine and metabolic responses to inflammation allow the organism to survive. However, in chronic inflammatory conditions, the inhibition of the hypothalamic–GH–IGF-1 axis contributes to the catabolic process, with skeletal muscle atrophy and cachexia. Here, we review the changes in pituitary GH secretion, IGF-1, and IGF-1 binding protein-3 (IGFBP-3), as well as the mechanism that mediated those responses. The contribution of GH and IGF-1 to muscle wasting during inflammation has also been analyzed.

## 1. Introduction

The physiological response to inflammation includes changes in the neuroendocrine system, which helps to combat the aggressor and favors the adaptation of the body to the new situation. This neuroendocrine response is an adaptive or stress response, which leads to the release of energy substrates from their storage in adipose tissue, liver, and skeletal muscle, in order to be consumed by the activated immune system [1].

Sepsis is a systemic inflammatory response induced by infection. Catabolism, body weight loss, growth impairment, muscle atrophy, and cachexia are complications of sepsis and chronic inflammatory diseases. The adverse effects of inflammation on growth and skeletal muscle mass can be due to several factors that help the organism to adapt in response to aggressions, such as (1) predominance of catabolic over anabolic hormones (cortisol, glucagon and adrenaline/insulin, IGF-1, gonadal steroids), (2) inflammatory mediators (cytokines, prostaglandins, nitric oxide), and (3) anorexia, poor nutrition, and/or malnutrition.

## 2. The Hypothalamic–GH–IGF-1 Axis in Inflammation

Growth hormone (GH) is secreted from the anterior pituitary in an episodic manner; it stimulates insulin-like growth factor-I (IGF-1) in the liver, but GH also has important direct actions on growth and metabolism. The hypothalamus regulates pituitary GH secretion by releasing two hormones; one is GH-releasing hormone (GHRH), which stimulates GH secretion, and the other is somatostatin, which inhibits it. Other peptide hormones from the periphery that also modulate GH secretion are IGF-1 released from the liver, which decreases GH secretion in a negative feedback loop, and ghrelin released from the stomach, which increases GH secretion.

The hypothalamic–GH–IGF-1 axis, similar to other endocrine axes, is modified by inflammation. Endotoxin or *E. Coli* lipopolysaccharide (LPS) injection has been shown to be an experimental model of the initial phase of sepsis induced by Gram-negative bacteria. IGF-1 secretion from the liver is very sensitive to inflammatory stimuli, since both serum concentration of IGF-1 and its synthesis in the liver decrease shortly after acute infection or endotoxin administration [2]. On the contrary, the effect of inflammation on pituitary GH secretion varies depending on the animal species analyzed as well as on the intensity and duration of inflammatory stimuli (Table 1).

In rodents, a low-dose intraperitoneal endotoxin injection increases pituitary GH mRNA and serum concentrations of GH, whereas it decreases circulating IGF-1 [3]. However, when the LPS dosage is increased, both circulating GH and IGF-1 decrease [2]. Taking into account that LPS directly stimulates GH release from pituitary cell cultures [3], the inhibitory effect of LPS on GH seems to be mediated by an indirect effect on the pituitary somatotroph cells. In addition, LPS injection at high but not at low dosages increased hypothalamic somatostatin expression [4], suggesting that the hypothalamic expression of somatostatin increases when circulating GH is decreased by endotoxin. Therefore, the inhibitory effect of acute inflammatory response can be mediated by the increased release of hypothalamic somatostatin. Indeed, an LPS-induced decrease in circulating GH can be prevented by a somatostatin antiserum [5].

In humans, acute LPS administration increases GH secretion, although it decreases circulating IGF-1 [6]. Discrepancies between data in humans and rodents can be due to the LPS dose, since authors injected 4 ng/kg to humans [6], whereas in rodents, the LPS dosages employed are between 5 µg/kg and 10 mg/kg (Table 1). Another possibility is that in the acute inflammatory process, the GH response is different depending on the species.

On the other hand, in chronic inflammatory conditions, such as experimental arthritis, plasma concentrations of IGF-1 and GH as well as pituitary GH mRNA are decreased [2,7,8]. In children with cystic fibrosis, juvenile idiopathic arthritis, or inflammatory bowel diseases, an alteration in both GH and IGF-1 secretion has also been observed, together with growth retardation [9,10]. Furthermore, critically ill patients during the acute phase of the illness have an increase in GH with a decrease in IGF-1 and in the main plasma IGF-1 binding protein-3 (IGFBP-3). However, in the chronic phase GH, IGF-1, and IGFBP-3 are decreased [11]. All these data indicate that in chronic inflammatory diseases, both humans and rodents show an inhibition of GH secretion.

### 2.1. Inflammation Induces GH Resistance

GH resistance is characterized by a decrease in the cells’ responsiveness to this hormone, causing a decline in circulating IGF-1 and its synthesis in the liver. Critically ill patients have increased GH secretion together with a decrease in circulating IGF-1 levels [14], suggesting GH resistance. Serum IGF-1 is reduced in the acute phase of critically ill patients with sepsis and was inversely related with the severity of the sepsis [15]. Similarly, as mentioned above, endotoxin administration at low doses increased GH secretion, whereas it decreased serum concentration of IGF-1 and IGFBP-3 as well as their expression in rat liver [16]. Therefore, in all the animal species tested, acute endotoxin administration induces GH resistance in the liver.

GH resistance is also developed in catabolic illness such as malnutrition, hepatic disorders, trauma and major surgery, chronic renal failure, as well as in states of severe inflammation such as ulcerative colitis and sepsis [17,18,19]. Although this response leads to increased muscle catabolism and poor wound healing, it allows the availability of free amino acids to be used for the synthesis of acute-phase response proteins and as neoglycogenic precursors [20].

Mechanistically, desensitization to GH can occur at the GH receptor (GHR) level through down-regulation of GH receptor abundance. In this sense, endotoxin administration has been shown to down-regulate hepatic GHR mRNA and protein [12,21]. In addition, a post-transcriptional proteolytic cleavage of GHR has been reported by Wang et al. [13], showing another mechanism for LPS-induced decrease in hepatic GHR abundance.

GH resistance may also take place at the post-receptor level involving intracellular GH-dependent signaling pathways. Binding of GH to its receptor activates Janus kinase 2 (JAK-2) and Lyn kinase (Lck/Yes), initiating the activation of the main groups of signaling molecules that mediate GH actions. These include the signal transducer and activator of transcription (STAT) pathway, the mitogen-activated protein kinase (MAPK) pathway, and the phosphoinositide-3 kinase (PI3K) pathway [22]. Post-receptor GH resistance is associated with the suppressor of cytokine signaling proteins (SOCS), particularly SOCS1 and SOCS3. These are negative regulators of the Janus kinase JAK2/STAT pathway [23]. These mechanisms are activated by inflammatory cytokines in LPS-induced sepsis [24].

Pro-inflammatory cytokines such as tumor necrosis factor (TNF)-α, interleukin (IL)-1β, and IL-6 play very important roles in inducing GH resistance. In vitro studies have shown that both TNF-α and IL-1β decrease liver GHR expression, downregulating GH response [17,25,26]. In addition, in vivo studies demonstrated that exogenous IL-6, TNF-α, and IL-1β inhibit GH signaling [25,26]. Another mechanism proposed [19,26] was that TNF-α and IL-1β act primarily on GHR abundance and IL-6 acts primarily on SOCS3.

Fasting, similar to the acute phase of inflammation, increases GH secretion but induces GH resistance in the liver, which leads to a decrease in circulating IGF-1 levels. It has been reported that liver IGF-1 secretion, in addition to GH, requires nutrients, specifically dietary proteins and amino acids [27]. The different responses of the two hormones to acute inflammation allows the coordination of their metabolic responses. On one hand, the increase in GH activates the release of free fatty acids and glucose to preserve fuel to cells. On the other hand, the decrease in circulating IGF-1 leads to the inhibition of skeletal muscle protein synthesis and bone growth.

Taking into account that the acute phase of inflammation is associated with anorexia, an inflammation-induced decrease in circulating IGF-1 could be secondary to the decrease in food intake. Although IGF-1 synthesis in the liver is decreased after fasting overnight, fasting is not the only cause of inflammation-induced inhibition of liver IGF-1, since LPS administration to fasted rats is still able to decrease body weight as well as IGF-1 levels in serum and its expression in the liver [28]. Nevertheless, an important relationship between malnutrition and inflammation seems to exist. Not only does inflammation induce anorexia and decrease in food intake, it has also been recently reported that arginine administration to fasted mice is able to prevent GH resistance and to increase IGF-1 and body weight by decreasing the Toll-like receptor 4-mediated inflammatory pathway [29].

In contrast to acute inflammation, GH has positive actions on some of the effects of chronic inflammatory diseases. GH administration is able to ameliorate growth impairment induced by polyarticular juvenile idiopathic arthritis or cystic fibrosis [30]. Furthermore, rhGH treatment, at relatively high doses, is able to overcome the relative GH resistance and ameliorate the growth delay in juvenile idiopathic arthritis [31]. In addition, in experimental arthritis, rhGH increases body weight as well as hepatic and serum concentrations of IGF-1 [32].

### 2.2. Inflammation Modifies Circulating IGF-Binding Protein-3

IGF-binding proteins (IGFBPs) are seven proteins that bind the two IGFs. IGFBP-1, 2, 3, and 5 are synthetized in the liver and are secreted to the plasma. Among these proteins, IGFBP-3 is the most abundant and the main carrier of IGF-1 in plasma, which is why it is one of the key proteins in the IGF-1-IGFBPs system. Circulating IGFBP-3 together with the acid-labile subunit (ALS) form a high molecular weight complex. Since that complex cannot leave the blood vessel and interact with its receptor, IGFBP-3 binding to IGF-1 in plasma decreases IGF-1 bioavailability. However, circulating IGFBP-3 notably increases the IGF-1 half-life from minutes to almost 16 h [33]. On the other hand, IGFBP-3 synthetized in tissue regulates IGF-1 availability in tissue by modulating IGF binding to its receptors. In addition, local IGFBP-3 can have several actions on the cells that are IGF-1 independent. These include cell growth regulation, survival, and apoptosis, by interacting with nuclear receptors in the target cells; for a review, see [34].

Sepsis in humans and experimental sepsis in animals decrease both circulating IGF-1 and IGFBP-3 [16,35]. Similarly, in children with systemic inflammation or critical illnesses, a decrease in both circulating IGF-1 and IGFBP-3 levels has been reported [36,37]. Circulating IGF-1 and IGFBP-3 are also decreased in patients with Crohn’s disease or in experimental colitis [38,39], where the inflammatory markers correlated with both proteins.

However, in children with dwarfism secondary to low GH secretion, serum IGF-1 levels are below the normal range, although IGFBP-3 levels are not [40]. Circulating IGFBP-3 seems to be less affected by undernutrition than IGF-1, since in Crohn’s disease patients, there is correlation between body mass index and IGF-1 but not with IGFBP-3 [38]. In addition to modifications of IGFBP-3 synthesis in the liver, changes in the rate of IGFBP-3 proteolysis in plasma can also contribute to the plasma concentrations of IGFBP-3 levels. In this sense, increased IGFBP-3 proteolysis has been reported in physical stress conditions such as diabetes and after surgery [41,42,43]. However, endotoxin-induced decrease in circulating IGFBP-3 does not seem to be secondary to increase in its plasma proteolysis [3].

### 2.3. Inflammation Decreases IGF-1 and IGFBP-3 Synthesis in the Liver

Inflammation-induced decrease in the serum of IGF-1 and IGFBP-3 is secondary to an inhibition of their synthesis in the liver, since the liver is the main source of both proteins in plasma. Accordingly, decreases in liver IGF-1 and IGFBP-3 mRNA have been reported after endotoxin administration and in experimental colitis [3,39].

In addition to inducing GH resistance, the fact that cytokines are able to inhibit IGF-1 expression in hepatocyte cultures [44,45] indicates that inflammation is able to directly down-regulate hepatocyte IGF-1 synthesis by a GH-independent mechanism. In this sense, it has been reported that hepatocytes express Toll-like receptors, and these cells respond to LPS through a TLR4 response pathway [46]. LPS and cytokines stimulate the expression of inducible nitric oxide synthase (iNOS) and consequently, the release of nitric oxide (NO). The increased release of NO by hepatocyte seems to play an important role in inflammation-induced downregulation of liver IGF-1, because iNOS inhibitors are able to prevent the inhibitory effect of LPS on IGF-1 both in vivo and in primary hepatocyte cultures [44,47].

**Figure 1:**
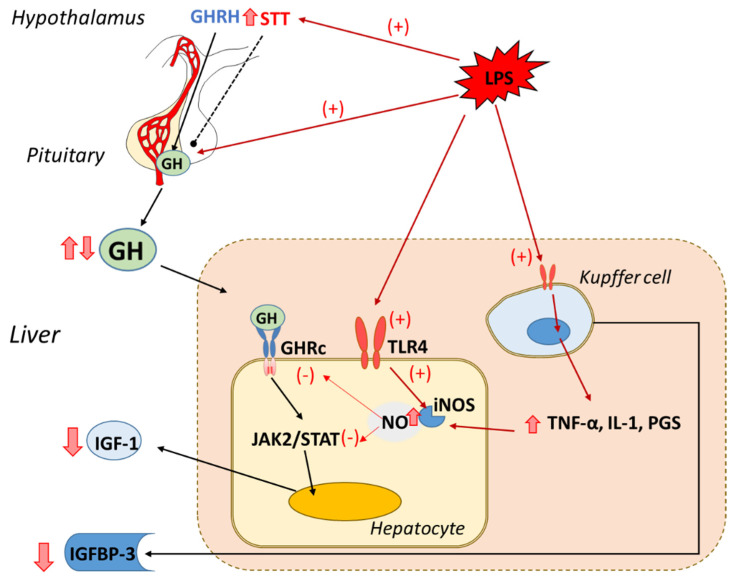
Effect of endotoxin or lipopolysaccharide (LPS) administration on the hypothalamic–GH–IGF-1 axis. At low dosage, LPS increased pituitary GH release, but with greater LPS dosages, an increase in hypothalamic somatostatin and a decrease in plasma GH is observed. Endotoxin directly decreased IGF-1 synthesis by hepatocytes through TLR4 activation, inducible nitric oxide synthase (iNOS) induction, and nitric oxide NO release. In addition, Kupffer cells, when stimulated by LPS, release inflammatory mediators (TNF-α, interleukins, and PGS), which further downregulate IGF-1 synthesis in hepatocytes. Whereas most IGF-1 is produced by hepatocytes, IGFBP-3 is mainly synthetized by Kupffer cells [49,50], and its synthesis is decreased by LPS. Red solid arrows indicate stimulatory (+) or inhibitory (−) effects of LPS. Black solid arrows indicate stimulatory and black dotted line indicates inhibitory effects of the hormones implicated in the GH-IGF-1 axis. Red border arrows indicate increased (upward) and decreased (downward) levels of the molecules/mediators in response to LPS.

Kupffer cells are key elements in the liver’s response to endotoxin-releasing cytokines and other mediators of the acute phase response. Indeed, Kupffer cell inactivation by gadolinium administration prevents the inhibitory effect of LPS injection on IGF-1, whereas the increase in ACTH and glucocorticoid secretion was not affected [48]. Furthermore, when primary hepatocytes were cocultured with Kupffer cells, the inhibitory effect of LPS on IGF-1 was higher than in the hepatocyte cultured alone [44]. Those data indicate an important role of Kupffer cells releasing inflammatory mediators when stimulated with bacterial endotoxin (Figure 1).

## 3. The Essential Role of Inflammation and IGF-1 on Muscle Mass

### 3.1. Inflammation-Induced Muscle Wasting

The endocrine response to acute inflammation plays an important role in maintaining the energy supply to the immune system and essential organs by increasing catabolism and decreasing anabolism. Although the authors were not able to find an increase in hepatic glucose production or in whole-body lipolysis in burned humans with sepsis, they observed an increase in muscle proteolysis [51]. Similarly, muscle wasting in critically ill patients with sepsis within the first week can lead to a 20% decrease in muscle mass [52,53]. Free amino acids from skeletal muscle can be converted into glucose in the liver or be used as fuel for other cell types. The metabolic results of these responses include an increase in glucose released from the liver, which is aimed at fulfilling the needs of the immune and nervous systems.

Therefore, the skeletal muscle not only preserves locomotion but also serves as a store of energy in the form of proteins and amino acids, which can be released to circulation in an emergency or stress [54]. The skeletal muscles affected by inflammation are mainly limb muscles. As mentioned above, inflammation-induced muscle atrophy is secondary to an increase in the activity of the muscle proteolysis pathways [55]. In both the acute phase of inflammation and chronic inflammatory diseases, there is an activation of the two proteolytic pathways, the ubiquitin–proteasome system and autophagy [56,57].

The catabolic response is secondary to the decrease in insulin, IGF-1, and gonadal steroid hormone secretion and to the increase in glucagon, cortisol, and GH secretion. Taking into account that IGF-1 has an anabolic effect on muscle protein and that glucocorticoids have a catabolic effect, it is not surprising that inflammation induces skeletal muscle proteolysis. In addition to the role of hormones, the release of pro-inflammatory cytokines and other mediators of inflammation have the ability to activate muscle proteolysis. Finally, as observed in liver IGF-1 regulation, amino acid administration to voluntary humans in the acute phase of inflammation decreased muscle proteolysis without modifying the inflammatory response to endotoxin [58].

### 3.2. The Antagonistic Effects of IGF-1 and IGFBP-3 on Skeletal Muscle Mass during Inflammation

IGF-1 is one of the most important regulators of muscle mass, and it increases muscle mass and strength [59]. This hormone induces muscle hyperplasia and hypertrophy by decreasing muscle proteolysis and increasing cell proliferation and differentiation [60,61]. IGF-1 can act on the skeletal muscle in an endocrine or paracrine manner, depending on its origin: the liver or whether it was locally synthetized in the muscle. The systemic administration of IGF-1 is able to ameliorate the effects of chronic inflammation on body and skeletal muscle weights by decreasing muscle proteolysis and increasing the expression of the myogenic transcription factors involved in myogenesis and proliferation [62]. However, although selective deletion of the IGF-1 gene in the liver of mice decreases circulating IGF-1 to 20%, those mice exhibit normal growth [63,64]. These data suggests that IGF-1 synthetized locally plays a role as important as or even more important than systemic IGF-1.

Sepsis and endotoxin administration also decrease IGF-1 expression in skeletal muscle [65,66,67]. Furthermore, local IGF-1 administration is able to prevent sepsis-induced skeletal muscle wasting [67]. On the contrary to IGF-1 expression, the expression of IGFBP-3 in skeletal muscle is upregulated by LPS administration and also by chronic inflammation induced by experimental arthritis [68,69]. Those data suggest that during inflammation, IGF-1 and IGFBP-3 have different regulation in the skeletal muscle.

IGFBP-3 expression is regulated in a different way depending on the tissue or organ analyzed. Whereas in the liver GH is the main regulator [70], in the small intestine, enterocytes IGF-1 increases IGFBP-3 mRNA [71]. On the contrary, IGF-1 administration decreased the expression of this binding protein in the gastrocnemius muscle [72]. In vivo, the effects of GH and IGF-1 on skeletal muscle are probably modulated by interactions with various IGFBPs.

Although IGFBP-3 contribution to muscle wasting during inflammation is not well known, several data indicate that an excess of this protein can induce muscle atrophy. IGFBP-3 is one of the two main IGFBPs expressed in skeletal muscle; it has a nuclear localization sequence (NLS) allowing them to directly modulate gene transcription in myoblast [73]. IGFBP-3 has been reported to be a binding partner of retinoic receptors (RXR and RAR), where these receptors are essential for IGFBP-3-induced apoptosis in cancer cell lines, and for the inhibitory effect of IGFBP-3 on preadipocyte maturation [74]. In addition to the fact that IGFBP-3 prevents IGF-1 binding to its receptor, IGFBP-3 is able to decrease myoblast proliferation and differentiation as well as the expression of the muscle regulatory factors MyoD and myogenin in absence of IGF-1 [75,76]. Accordingly, IGFBP-3 blockade increases DNA synthesis in myoblast [77]. Muscle proteolysis has also been reported to be increased by IGFBP-3 via suppression of IGF-1/PI3K/AKT signaling in myotube cultured cells [75].

**Figure 2:**
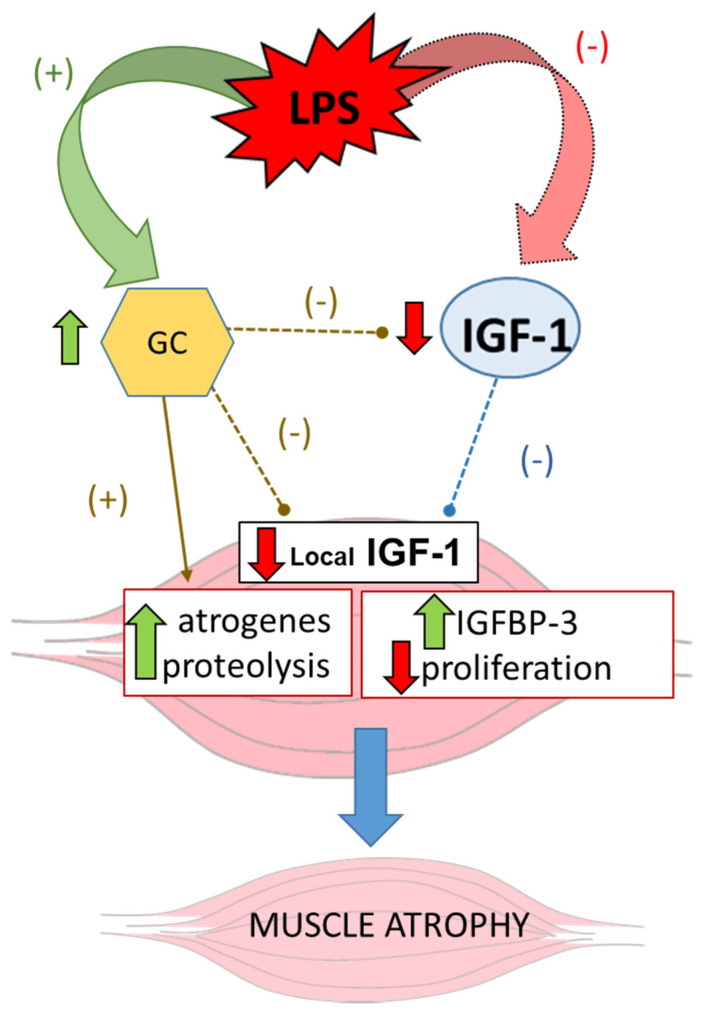
Role of IGF-1 in inflammation-induced muscle atrophy. The decreased secretion of GH and/or GH resistance, together with the increased release of glucocorticoids (+) induce downregulation of plasma and muscle IGF-1 (−), and the increase in muscle IGFBP-3. Low plasma IGF-1 and high glucocorticoid levels increase atrogenes expression (MuRF-1 and atrogin-1), activate muscle proteolysis by the ubiquitin–proteasome system, and decrease protein synthesis. The increased IGFBP-3 expression together with the low IGF-1 levels in muscles decrease muscle proliferation. Thin arrows indicate stimulatory (+) and dotted ones inhibitory (−) effects of GC (green) and IGF-1 (blue) on muscle. Thick arrows indicate stimulation (green upward arrows) and inhibition (red downward arrows) of molecules/processes in muscle.

In addition to the inhibition of the GH–IGF-1 axis, another possible factor mediating skeletal muscle wasting during inflammation is glucocorticoids. The inhibitory effect of glucocorticoids on plasma and muscle IGF-1 and its adverse effect on muscle mass are well known. Acute inflammation activated the hypothalamic–pituitary–adrenal axis, increasing the secretion of glucocorticoids [57], and usually, patients with chronic inflammatory illness are treated with corticoids, which further decrease muscle mass. All these data suggest that during inflammation, the decrease in serum and muscle IGF-1, together with the increase in muscle IGFBP-3, are associated with the increase in muscle proteolysis and cachexia (Figure 2).

Local IGFBP-3 has been shown to be an inhibitor of cell growth, and it induces apoptosis by an IGF-1-independent mechanism in different tissues [78]. A decrease in myogenic cell proliferation by local IGFBP-3 has also been reported [76]. Not only local IGFBP-3 synthetized in skeletal muscle can be associated with muscle wasting and cachexia in sepsis or in other inflammatory diseases, but also an excess of systemic IGFBP-3 can contribute to muscle atrophy. Several types of cancer are associated with muscle wasting and cachexia, and this progressive cachexia can result in death by respiratory failure and infection [79]. In this sense, Huang et al. [75] reported that a large of amount of IGFBP-3 is released by the cells of pancreatic ductal adenocarcinoma. Furthermore, medium from these pancreatic cancer cells in culture is able to induce muscle atrophy, and this effect can be blocked by IGFBP-3 antibody neutralization [75].

## 4. Conclusions

Inflammation activates catabolism, leading to cachexia that increases morbidity and mortality. These catabolic pathways include GH resistance as well as a decrease in liver IGF-1 and IGFBP-3 synthesis and their plasma levels. Endotoxin is also able to directly act on the skeletal muscle decreasing local IGF-1. Taking into account that IGF-1 is a hormone that prevents muscle atrophy and increases muscle mass, the down-regulation of circulating and muscle IGF-1 plays an important role in inflammation-induced cachexia. In addition, the increased IGFBP-3 expression in skeletal muscle also can contribute to muscle wasting by inducing apoptosis and inhibition of cell proliferation.

The possible treatment with IGF-1 for muscle-wasting conditions remains an important research challenge.

## Figures and Tables

**Table 1:** Effect of different inflammatory stimuli on pituitary GH secretion. Upward arrows indicate increased and downward arrows decreased pituitary GH secretion in response to the inflammatory stimuli.

Reference	Inflammatory Stimuli	Specie/Cell Culture	Pituitary GH Secretion (↑/↓)
[3]	Low LPS doses (5, 10, 50 and 100 µg/kg, i.p.)	Rodents	↑
[2,3,12,13]	High LPS doses (100, 250, 500 and 1000 microg/kg, i.p.)	Rodents	↓
[6]	LPS (4 ng/kg, i.v.)	Humans	↑
[3]	LPS (0.1 and 10 ng/mL)	Pituitary cell culture (rodents)	↑
[2,7,8]	Experimental arthritis	Rodents	↓
[9,10]	Cystic fibrosis, inflammatory bowel disease	Humans	↓
[11]	Critical ill patients:	Humans	
	acute phase		↑
	chronic phase		↓
